# Do dietary intakes influence the rate of decline in anti-Mullerian hormone among eumenorrheic women? A population-based prospective investigation

**DOI:** 10.1186/s12937-019-0508-5

**Published:** 2019-12-02

**Authors:** Nazanin Moslehi, Parvin Mirmiran, Fereidoun Azizi, Fahimeh Ramezani Tehrani

**Affiliations:** 1 Nutrition and Endocrine Research Center, Research Institute for Endocrine Sciences, Shahid Beheshti University of Medical Sciences, Tehran, Iran; 2 Department of Clinical Nutrition and Dietetics, Faculty of Nutrition and Food Technology, National Nutrition and Food Technology Research Institute, Shahid Beheshti University of Medical Sciences, Tehran, Iran; 3 Endocrine Research Center, Research Institute for Endocrine Sciences, Shahid Beheshti University of Medical Sciences, Tehran, Iran; 4 Reproductive Endocrinology Research Center, Research Institute for Endocrine Sciences, Shahid Beheshti University of Medical Sciences, Tehran, Iran

**Keywords:** Anti-Müllerian hormone, Ovarian reserve, Diet, Dairy

## Abstract

**Background:**

Dietary intakes are suggested to affect age at menopause but associations between dietary factors and ovarian reserve reduction have not yet been investigated. We aimed to examine dietary intakes in relation to the rate of decline in anti-Mullerian hormone (AMH), an indicator of ovarian reserve, in a generally healthy cohort of women.

**Methods:**

This prospective investigation was conducted among 227 eumenorrheic women, aged 20–50 years, from the Tehran Lipid and Glucose study, who were followed over a mean of 16 years. AMH was measured twice, at baseline and the 5th follow-up examination cycle, and yearly rate of decline in AMH was calculated. Rapid decline in AMH was defined as the annual percent change AMH > 5.9%/year based on tertile 3 of the variable. Average usual dietary intakes were estimated using the food frequency questionnaires administered at the second, third, and the fourth follow-up examinations. After adjusting for potential covariates, the association between dietary factors and both risk of rapid decline in AMH and also annual percent decline of AMH (as a continuous variable) were examined using logistic regression and the Spearman correlation, respectively.

**Results:**

The baseline age of the participants and the median rate of decline in AMH were 37.2 years and was 5.7% yearly, respectively. The odds of rapid decline in AMH was reduced by 47% for dairy products (95% CIs = 0.36, 0.79; *p* = 0.002), 38% for milk (95% CIs = 0.41, 0.93; *p* = 0.020), and 36% for fermented dairy (95% CIs = 0.45, 0.93, *p* = 0.018) per one standard deviation (SD) increase in their dietary intakes. The odds of rapid decline in AMH was significantly reduced with higher intakes of fat, carbohydrate, protein, and calcium intakes from dairy sources, lactose and galactose. Annual rate of AMH decline was inversely correlated with dairy products, milk, fermented dairy, fruits, dairy carbohydrate, dairy fat, dairy protein, total calcium and dairy calcium, lactose and galactose, and positively correlated with organ meats.

**Conclusion:**

Dairy foods consumption may reduce the rate of AMH decline in regularly menstruating women. Life style modification in terms of dietary advice may be considered as a preventive strategy for reduction in the rate of ovarian reserve loss.

## Introduction

The numbers of ovarian follicles represent the reproductive age of women and their reproductive capacity [1]. Anti-mullerian hormone (AMH), now suggested as the best indirect measures of ovarian reserve [1], has different implications in clinical practice, from diagnosis of women with polycystic ovarian syndrome (PCOS) or those with diminished ovarian reserve to prediction of infertility treatment success rates and time to menopause [2–4]. Generally, AMH decreases gradually as the follicular pool declines and becomes undetectable when menopause occurs [1]. However, AMH concentrations in women of similar age are not identical and acceleration of their decline over time highly differ inter-individually [5–7]. The rate of AMH decline was suggested as a predictor of time to menopause, independent of AMH baseline value and age [5]. This rate also suggested as a risk factor for cardiovascular and coronary heart disease in women independent of metabolic and menopausal risk factors [8, 9].

In addition to age and genetics, the strongest predictors of ovarian reserve, lifestyle and environmental factors seem to modify the recruitment of follicles and/or follicular atresia [10–12]. Some, but not all, epidemiological studies found significant associations between some dietary factors and timing of menopause [13]. Despite the heterogeneity in dietary factors and findings across studies, they indirectly underscore the possibility that nutrition and dietary intakes may influence ovarian reserve. Recently a cross-sectional study examined the associations of macronutrients, fiber, and glycemic index with AMH, and reported an inverse association between percentage of energy from dietary fat and AMH [14]. Thus far no prospective study has been conducted to investigate the influence of dietary factors on the rate of AMH decline, and the question as to whether ovarian aging can be altered by dietary factors remains to be answered. Therefore, the purpose of present study was to examine the prospective associations of dietary factors with the rate of AMH decline in a general cohort of women, participated in a population based study.

## Material and methods

### Participants

**Fig. 1:**
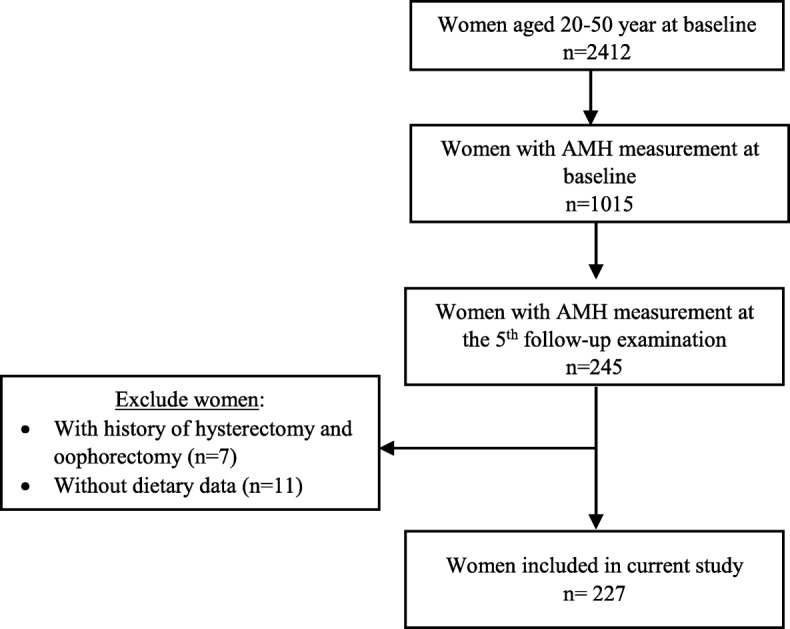
Study flowchart

Participants of this prospective study were selected from the Tehran Lipid and Glucose Study (TLGS), an ongoing, population based study being conducted in Tehran, Iran; the TLGS was started in 1999 with 15,005 individual aged ≥3 years, selected randomly from residents of district 13 of Tehran, the capital of Iran, after which the participants are re-examined every 3 years [15, 16]. Five follow-up examination cycles have been completed in 2001–2005, 2005–2008, 2009–2011, 2011–2015, and 2015–2018. All baseline and follow-up examinations have been taken place at the TLGS Unit. AMH was measured at baseline for all women, aged 20–50 years, who met the eligibility criteria of having regular and predictable menstrual cycles, having proven natural fertility, and no history of endocrine disorders, hysterectomy, oophorectomy or any other kind of ovarian surgery (*n* = 1015) [17]. Due to financial constraints, a subsample of 245 women were randomly selected to measure AMH in the 5th follow-up examination. By excluding 7 women with history of hysterectomy and oophorectomy during follow-ups and 11 women with no dietary intake information, finally 227 women remained for current analysis (Fig. 1). Socio-demographic and anthropometric characteristics of women included in this study were not significantly different from those who did not include, except for age which was significantly higher in those included in the study (Additional file 1: Table S1). The ethics committee of the Research Institute for Endocrine Sciences of Shahid Beheshti University of Medical Sciences approved the study protocol, and written informed consent was obtained from all participants.

### Demographic and reproductive information

Demographic and reproductive data on age, education, occupation, smoking, age at menarche, menstrual cycle status, and marital status have been collected at baseline and through follow-ups, during face to face interviews by trained physicians.

Weight and height were measured with light clothing, and without shoes to the nearest 100 g and 0.1 cm, respectively. Body mass index (BMI) was calculated as weight (kg)/height^2^ (m^2^). Based on standard BMI cut-offs established by world health organization (WHO), 3 BMI categories were defined as normal weight (BMI < 25 kg/m^2^), overweight (25–29.9 kg/m^2^), and obese (BMI ≥ 30 kg/m^2^, [18]). BMI change during the follow-up was determined as the difference between BMI measured at the 5th follow-up examination and baseline.

### Dietary information

Dietary intakes were determined using a 168-item food frequency questionnaires during face-to-face interview by trained nutritionists. Reliability and validity of the FFQ to assess nutrients and food group intakes have been previously documented [19, 20]. Frequency of consumption of each item food, and amount of consumption according to a predefined portion sized during the past year were obtained from participants. In this study, food items were categorized into 32 food groups (Additional file 2: Table S2), and 4 food items (soy, eggs, honey, and soft drinks) were considered individually. Tomatoes was also considered individually as a subgroups of vegetables.

Daily nutrients intakes were determined using both the traditional Iranian and the United States Department of Agriculture (USDA) food composition tables [21, 22]. For the current study, dietary intakes of macronutrients, saturated fatty acids (SFA), mono-unsaturated fatty acids (MUFA), poly-unsaturated fatty acid (PUFA), fiber, and calcium were examined.

To reduce within person variability in dietary intakes during the follow-up, mean scores were determined from the dietary data of the second (2005–2008), third (2009–2011), and the fourth (2012–2015) follow-up examinations.

### Anti-Müllerian hormone measurements

Blood samples were collected at baseline and each follow-up visit by trained staffs and stored at −80C for future use. AMH was measured at the time of recruitment and the 5th follow-up examination cycle using stored samples by the two-site enzyme immunoassay (EIA) method using Gen II kit (Beckman Coulter, Inc.CA, USA) and the Sunrise ELISA reader (Tecan Co. Salzburg, Austria). AMH Gen II controls A79766 were used at two levels of concentration to monitor accuracy of assay. The intra- and inter-assay CVs were 3.5 and 3.8% respectively. Lower limit of sensitivity was 0.08 ng/ml.

The rate of decline in AMH was calculated by the unit change from baseline to the 5th follow-up examination cycle divided by the baseline value, and was expressed as percentage. To estimate the annual decline in AMH, the percent decline in AMH was divided by the time interval (in years) between baseline and the 5th follow-up.

### Statistical analysis

Characteristics of participants were reported using descriptive statistics as mean ± standard deviation (SD) for normally distributed variables, median (interquartile range, IQR) for non-normally distributed variables, and number (%) for categorical variables.

The participants were divided into three groups based on tertiles of annual decline in AMH, after which women in tertile 3 with decline in AMH > 5.9%/year were defined as the rapid decline group. Since there is no normative value to define the rapid decline in AMH based on its yearly decline, we decided to use the last tertile of annual decline in AMH as a cut-off to define individuals with rapid rate of decline. Characteristics of the participants by tertiles of annual decline in AMH were examined using ANOVA test for normally distributed variables and Kruskal-Wallis test for non-normally distributed variables. Associations between dietary exposures and risk of rapid decline in AMH were examined using logistic regression in the unadjusted and adjusted models for baseline age, BMI, and energy intakes. Data are presented as odds ratios (95% confidence intervals [CIs]) per one SD increase in intakes of food groups and nutrients. Age, age at menarche, baseline BMI, education, and smoking were selected as potential covariates based on previous studies [23–25]. However, age at menarche and education were not considered for inclusion because they showed no significant associations with AMH and adding to the final model did not substantially change the odds estimates. Smoking was also not included because of the low number of smokers (*n* = 8) among the participant.

Correlations between dietary intakes and the annual rate of decline in AMH as a continuous variable were also examined. The annual decline in AMH was highly skewed and transformations could not improve its distribution. Therefore, associations between the annual rate of AMH decline and dietary intakes were examined using Partial Spearman Correlation after adjusting for baseline age, BMI, and energy intakes. Analyses were performed in SPSS software, version 20 (IBM Corp, Armonk, NY) and *p*-values ≤0.05 were assumed to be significant statistically.

## Results

Mean age at baseline and duration of follow-up were 37.2 ± 6.33 and 16.3 ± 1.02 years, respectively. Characteristics of participants are reported in Table 1. The majority of women were non-smokers (96.5%), and were overweight (45.8%) at baseline. The median (IQR) of AMH at baseline was 0.55(0.22, 1.54) ng/ml, and the median rate of decline in AMH during the follow-up was 5.68% per year. Age (P-ANOVA = 0.003) and baseline AMH concentrations (P- Kruskal Wallis = 0.004) were significantly different across tertiles of annual decline in AMH (Table 1).

**Table 1:** Characteristics of study participants^1^

Characteristics	Total	Annual decline in AMH			*P*-value^2^
		Lowest tertile	Middle tertile	Highest tertile	
Number	227	76	76	75	–
Follow-up time (years)	16.3 ± 1.02	16.9 ± 1.17	16.4 ± 0.84	15.6 ± 0.48	–
Baseline demographic					
Age (years)	37.2 ± 6.33	35.2 ± 6.58	38.3 ± 6.17	38.2 ± 5.82	0.003
Housewife (n(%))	183 (80.6)	61 (80.3)	64 (84.2)	58 (77.3)	0.562
Education > 12 years (n(%))^3^	132 (58.1)	44 (59.5)	49 (65.3)	39 (53.4)	0.337
Never smokers (n(%))	219 (96.5)	75 (98.7)	72 (95.0)	72 (96.0)	0.546
Anthropometric					
Baseline BMI (kg/m^2^)^4^	26.9 ± 4.3	26.3 ± 4.37	27.1 ± 3.99	27.3 ± 4.59	0.319
Baseline BMI categories^4^					
< 25 (n(%))	74 (32.6)	29 (38.2)	18 (24)	27 (36.5)	0.226
25–29.9 (n(%))	104 (45.8)	34 (44.7)	41 (54.7)	29 (39.2)	
≥ 30 (n(%))	47 (20.7)	13 (17.1)	16 (21.3)	18 (24.3)	
BMI change during follow-up (kg/m^2^)^5^	2.77 ± 3.72	2.52 ± 3.10	2.59 ± 3.43	3.19 ± 4.49	0.511
Reproductive					
Age at menarche (years)^6^	13.5 ± 1.37	13.4 ± 1.51	13.4 ± 1.26	13.7 ± 1.34	0.447
Baseline AMH (ng/ml)	0.55 (0.22, 1.54)	0.41 (0.12, 1.60)	0.50 (0.16, 0.91)	0.72 (0.35, 1.95)	0.004
Annual decline in AMH^7^ (%)	5.68 (4.93, 6.04)	4.42 (3.33, 4.96)	5.68 (5.52, 5.84)	6.16 (6.04, 6.36)	< 0.001
Dietary intakes					
Energy (kcal/day)	2274 ± 688	2328 ± 676	2225 ± 654	2268 ± 737	0.653
Carbohydrate (% of energy)	58.1 ± 5.94	57.6 ± 5.16	58.3 ± 6.77	58.3 ± 5.84	0.691
Fat (% of energy)	30.9 ± 5.26	31.1 ± 4.78	31.1 ± 5.35	30.4 ± 5.66	0.676
Protein (% of energy)	13.5 ± 2.16	13.6 ± 1.99	13.5 ± 2.21	13.5 ± 2.30	0.922

^1^Data are presented as mean ± standard deviation (SD), median (quartile 1, quartile 4), and Number (%). ^2^Based on ANOVA test for normally distributed variables and Kruskal-Wallis test for non-normally distributed variables. ^3^Available for *n* = 222. ^4^Available for *n* = 225. ^5^Available for *n* = 204. ^6^Available for *n* = 226. ^7^Calculated by the unit change from baseline to the 5th follow-up examination cycle divided by the baseline value

Odds of rapid reduction in AMH in relation to food groups are shown in Table 2. Higher intakes of dairy products were associated with lower odds of rapid reduction in AMH in both the unadjusted and adjusted models. After adjusting for baseline age, BMI, and energy intake, odds of rapid decline in AMH was significantly reduced by 47% (95% CIs = 21–64%), 38% (95%CIs = 7–59), and 36% (95%CIs = 7–55%) respectively per one SD increase in daily intakes of total dairy products (SD = 235 g), milk (SD = 146 g), and fermented dairy (SD = 156 g). No significant association was observed between the other food groups and odds of rapid reduction in AMH.

**Table 2:** Odds ratios (OR) and 95% confidence intervals (CIs) of rapid rate decline in AMH per one standard deviation (SDs) increases of daily food groups intakes

Daily intakes per 1-SDs (g/day)	Unadjusted			Adjusted^1^		
	OR	95%CIs	P	OR	95%CIs	P
Grain	1.18	0.90, 1.55	0.241	1.25	0.90, 1.74	0.188
Legumes	1.13	0.86, 1.48	0.373	1.22	0.90, 1.65	0.191
Soy	0.83	0.73, 1.29	0.825	0.98	0.71, 1.34	0.879
Total meat	0.88	0.63, 1.23	0.462	0.82	0.56, 1.19	0.290
Beef-lamb	0.92	0.65, 1.31	0.652	0.91	0.64, 1.29	0.594
Poultry	0.82	0.55, 1.22	0.334	0.73	0.46, 1.17	0.193
Fish	1.32	1.01, 1.72	0.046	1.31	1.00, 1.72	0.053
Tuna-fish	1.13	0.86, 1.47	0.383	1.14	0.86, 1.50	0.356
Organ meats	1.00	0.76, 1.32	0.985	1.01	0.77, 1.33	0.935
Egg	1.20	0.92, 1.58	0.181	1.30	0.97, 1.74	0.076
Dairy products	0.63	0.45, 0.88	0.006	0.53	0.36, 0.79	0.002
Milk	0.64	0.43, 0.93	0.021	0.62	0.41, 0.93	0.020
Fermented dairy	0.73	0.53, 0.99	0.044	0.64	0.45, 0.93	0.018
Vegetables	1.09	0.83, 1.44	0.532	1.04	0.77, 1.42	0.784
Allium	0.98	0.74, 1.30	0.882	0.94	0.70, 1.26	0.653
Cruciferous	0.96	0.72, 1.28	0.778	0.99	0.74, 1.32	0.919
Potatoes	0.95	0.71, 1.26	0.706	0.99	0.73, 1.34	0.991
Green vegetables	1.10	0.84, 1.45	0.497	1.05	0.77, 1.42	0.779
Tomatoes	1.09	0.83, 1.43	0.544	1.05	0.79, 1.41	0.725
Yellow-orange vegetables	1.10	0.84, 1.43	0.508	1.08	0.81, 1.44	0.613
Fruits	0.88	0.65, 1.19	0.398	0.82	0.57, 1.18	0.277
Fruit juices	1.11	0.84, 1.46	0.464	1.09	0.83, 1.44	0.521
Dried fruits	1.10	0.84, 1.45	0.493	1.14	0.85, 1.53	0.378
Melons	1.09	0.84, 1.43	0.512	1.09	0.81, 1.46	0.571
Stone fruits	0.82	0.58, 1.17	0.282	0.79	0.54, 1.16	0.238
Citrus fruits	0.84	0.62, 1.14	0.261	0.79	0.56, 1.12	0.184
Berries	0.83	0.60, 1.14	0.247	0.79	0.55, 1.15	0.216
Nuts	1.04	0.78, 1.40	0.779	1.06	0.81, 1.40	0.650
Olive & olive oils	1.05	0.80, 1.39	0.707	1.05	0.80, 1.40	0.718
Oil-fats	0.94	0.71, 1.25	0.677	0.96	0.71, 1.28	0.758
Butter	0.87	0.58, 1.29	0.474	0.91	0.60, 1.38	0.645
Fast foods	1.18	0.90, 1.55	0.221	1.27	0.94, 1.70	0.117
Sweets and cakes	0.98	0.74, 1.30	0.888	1.00	0.73, 1.35	0.977
Honey	1.10	0.85, 1.44	0.469	1.13	0.86, 1.49	0.380
Soft drinks	0.98	0.73, 1.30	0.862	0.97	0.72, 1.30	0.843
Tea and coffee	1.16	0.88, 1.53	0.290	1.16	0.88, 1.53	0.307
Salty snacks	1.04	0.79, 1.37	0.770	1.04	0.79, 1.38	0.776

^1^Adjusted for baseline age and BMI, and energy intake

SDs: Grains = 150; Legumes = 35.6; Soy = 11.5; Total meat = 53.9; Beef-lamb = 37.0; Poultry = 37.4; Fish 6.91; Tuna-fish = 5.44; Organ-meats = 3.29; Egg = 10.19; Dairy products = 235; Milk = 146; Fermented dairy = 156; Vegetables = 172; Allium = 26.7; Cruciferous = 12.12; Potatoes = 16.8; Green vegetables = 85.5; Tomatoes = 71.1; Yellow- orange vegetables = 28.9; Fruits = 324; Fruit Juices = 87.7; Dried fruits = 8.87; Melons = 66.8; Stone fruits = 69.7; Citrus = 88.0; Berries = 45.8; Nuts = 10.7; Olive- olive oils = 9.72; Oil-fats = 11.6; Butter = 15.3; fast foods = 17; Sweet-cakes = 34.4; Honey = 4.89; Softdrink = 52.4; Tea-coffee = 556; Salty snacks = 24.1

**Fig. 2:**
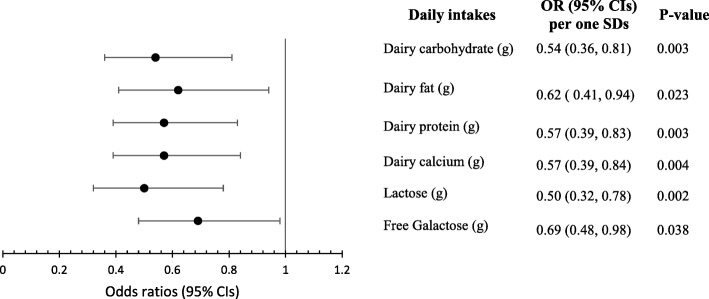
Odds ratios (95% CIs) of rapid decline in AMH per one SDs increases in dietary intakes of dairy constituents SDs: Dairy carbohydrate = 11.4; Dairy fat = 7.59; Dairy protein = 9.92; Dairy calcium = 283; lactose = 11.6; free galactose = 2.04 g/d

Dietary intakes of macronutrients, calcium, and fiber in relation to odds of rapid decline in AMH are reported in Table 3. Total intakes of macronutrients, SFA, MUFA, PUFA, fiber, and calcium were not significantly associated with odds of rapid decline in AMH. Due to significant association between diary intakes and AMH decline in this study, macronutrients and calcium intakes from dairy sources, and lactose and free galactose intakes in relation to decline in AMH were also examined. Results showed that higher intakes of carbohydrate, fat, protein and calcium from dairy sources had significantly reduced odds of rapid decline in AMH, even after adjusting for baseline age, BMI, and energy intake (Fig. 2). In addition, one SD increase in lactose (SD = 11.6 g) and free galactose (SD = 2.04 g) intakes predicted 50 and 31% less likelihood of rapid AMH decline respectively after adjusting for potential covariates (Fig. 2).

**Table 3:** Odds ratios (OR) and 95% confidence intervals (CIs) of rapid decline rate in AMH based on daily intakes of macronutrients, fiber, and calcium

Daily intakes per 1-SDs (g/day)	Unadjusted			Adjusted ^1^		
	OR	95%CIs	P	OR	95%CIs	P
Total carbohydrate	1.03	0.78, 1.35	0.851	1.04	0.99, 1.09	0.140
Total fat	0.93	0.70, 1.23	0.604	0.83	0.47, 1.48	0.526
Plant fat	1.02	0.78, 1.35	0.881	1.09	0.75, 1.59	0.657
Non-dairy animal fat	0.86	0.64, 1.17	0.333	0.82	0.57, 1.18	0.287
Total protein	0.94	0.70, 1.24	0.643	0.76	0.44, 1.31	0.319
Plant protein	1.13	0.86, 1.48	0.387	1.51	0.92, 2.49	0.107
Non-dairy animal protein	0.95	0.71, 1.28	0.750	0.91	0.65, 1.27	0.568
Saturated fatty acids	0.81	0.60, 1.10	0.174	0.65	0.40, 1.06	0.082
Mono-unsaturated fatty acids	0.98	0.74, 1.29	0.880	1.01	0.63, 1.63	0.962
Poly-unsaturated fatty acids	1.07	0.82, 1.41	0.611	1.20	0.81, 1.78	0.366
Trans fatty acids	0.77	0.51, 1.15	0.205	0.73	0.46, 1.16	0.181
Fiber	1.01	0.98, 1.03	0.516	1.03	0.98, 1.07	0.233
Calcium	0.86	0.64, 1.16	0.306	0.71	0.46, 1.11	0.133

^1^Adjusted for baseline of age and BMI, and energy intake

SDs: Total carbohydrate = 104; Total fat = 28.1; Plant fat = 19.1; Non-dairy animal fat = 11.2; Total protein = 28.3; Plant protein = 14.8; Non-dairy animal protein = 15.7; Saturated fatty acids = 9.61; Mono-unsaturated fatty acids = 9.68; Poly-unsaturated fatty acids = 7.60; Trans fatty acids = 3.53; Fiber = 11.4; Calcium = 375

Considering annual decline in AMH as a continuous variable showed that intakes of dairy products (rs = − 0.195, *p* = 0.004), fermented dairy (rs = − 0.191, *p* = 0.004), fruits (rs = − 0.133, *p* = 0.048), and berries (rs = − 0.137, *p* = 0.042) were inversely correlated and organ meats (rs = 0.163, *p* = 0.015) were directly correlated with rate of AMH reduction, independent of baseline age, BMI, and energy intakes. No significant correlation was observed between the other food groups and AMH rate change. Among nutrients, dairy carbohydrate (rs = − 0.179, *p* = 0.008), dairy fat (rs = − 0.174, *p* = 0.009), dairy protein (rs = − 0.192, 0.004), total calcium (rs = − 0.142, *p* = 0.035) and dairy calcium (rs = − 0.199, *p* = 0.003), lactose (rs = − 0.194, p = 0.004), and free galactose (rs = − 0.184, *p* = 0.006) were all inversely correlated with annual decline in AMH after adjusting for baseline age, BMI, and energy intake.

## Discussion

The present prospective study demonstrates that total dairy, milk and fermented dairy are inversely correlated with annual decline in AMH rate and lower odds of rapid decline in AMH, independent of baseline age, BMI, and energy intakes. In addition, fruits, berries, and total calcium were inversely, and organ meats were positively correlated with the AMH annual reduction but were not associated with odds of rapid decline in AMH. Higher dietary intakes of carbohydrates, fat, protein, and calcium from dairy sources, and free galactose and lactose were also associated with both lower annual reduction in AMH and the odds of its rapid decline. Other dietary factors were not associated with rate of AMH decline.

Concentrations of AMH decrease by reducing the pool of ovarian follicles from birth to menopause. However, there is large variations in the rate of follicular reduction among of women of similar age [5, 7]. Nutritional investigations indicate that food intakes may affect menopause, albeit dietary determinants of menopause remain mostly unknown because of inconsistency in findings, design, and nutritional factors examined across studies [13]. To date, few studies have directly investigated dietary intakes in relation to ovarian reserve [13]. A prospective study following the 259 women for 2 menstrual cycle found no significant association between dietary factors (macronutrients, sucrose, starch, total sugar and fiber) consumed during the menstrual cycle and AMH concentration [26]. Another study, a cross-sectional study of 296 women, aged 35–45 years, found a positive association of total carbohydrates and a negative association of total fat intake with AMH; of subtypes of fat, PUFA was also negatively associated with AMH, and other dietary factors examined, including protein, glycemic index and load, and fiber were not related to AMH [14]. Based on our findings, apparently the rate of ovarian follicular loss is not influenced by habitual intakes of macronutrients, either as absolute or relative to energy intake, and fiber; however, increasing carbohydrate, fat, and protein from dairy sources can reduce the rate of its decline.

No previous study has investigated the association between dairy products and ovarian reserve markers; two studies have however reported prospective associations of dairy intakes and occurrence of menopause [27, 28], with the former finding no significant association in 13,612 premenopausal women aged 35–65 years during a median follow-up of 5.8 years [27] and the other suggesting a delay in occurrence of menopause only in women aged < 51 years [28]. Findings of our study suggest that dairy food intakes may affect ovarian aging in premenopausal women. We did not categorize dairy intakes into low- and high- fat to reduce misclassification but an inverse association between dairy fat and AMH rate decline may suggest that higher intakes of fat from dairy sources may not be deleterious to ovarian follicles. Inverse association with AMH rate reduction was observed for all constituents of dairy examined in this study including macronutrients, calcium, and lactose. Although highly correlations between the nutrients make it difficult to investigate their independent associations with AMH rate decline, our findings demonstrate that a combination of nutrient and non-nutrient components of dairy products was involved. Consistent with our findings, dairy fat, dairy protein, and lactose reduced likelihood of menopause occurrence in women aged < 51 years based on the Carwile et al. study [28]. Despite lack of sufficient evidence for identification of the underlying mechanisms involved, it can be assumed that this lower rate of decline in AMH may indicate either the lower rate of follicular recruitments, by affecting ovulation or menstrual cycle, or higher production of AMH from granulosa cells with higher intakes of dairy foods. Previous findings of higher risk of sporadic anovulation with higher intakes of some types of dairy foods, including cream and yoghurt [29] and higher risk of anovulatory infertility with higher intakes of low fat [30] support the assumption that dairy products may slow follicular reduction by increasing anovulatory menstrual cycles. However, the association was not seen for total dairy intakes and high fat dairy. Whether or not dairy products influence the length of the menstrual cycle or the function of granulosa cells has not been investigated, yet.

Results from rodents fed high amount of galactose suggest a toxic ovarian effect for galactose [31, 32]. Early menopause in women with galactosemia and low activity of galactose-1-phosphat uridylyltransferase (GALT) enzyme also suggest ovarian toxicity due to galactose accumulation [33, 34]. Free galactose intakes from dietary sources including dairy products, fruits (peaches, cherries, melons, fig, kiwifruits), nuts (hazelnuts and almonds), and honey are low and in our study this amount of galactose was associated with reduced risk of accelerated declining in AMH. In addition, lactose, the main source of galactose intake in humans, also showed an inverse association with rate of decline in AMH. An inverse association was also reported for lactose intake and likelihood of menopause [28]. Furthermore, lactose intakes has been reported to slightly improve female fertility [35]. Based on the above, lactose and galactose at usual levels in diet of non-galactosemic women apparently improve ovarian function.

Higher intake of dietary antioxidant is proposed to alleviate apoptotic loss of primordial follicles due to oxidative stress [36, 37]. We found no significant association between fruits and vegetables, rich sources of antioxidants, and risk of rapid decline in AMH, either as total intakes or different subtypes. However, rate of decline in AMH was inversely correlated with total fruit and berries intakes. Fruit intakes were positively associated with later menopause in two studies [37, 38] while another found no significant association in this regard [27]. One study reported an inverse association for only green and yellow vegetables with menopause [36].

This is a first investigation attempting to determine habitual dietary intakes in relation to rate of ovarian reserve loss in reproductive eumenorrheic women; its population-based and prospective design are its most important strengths. A comprehensive examination of dietary factors, including food groups and nutrients is also among the strengths. This study however has some limitations; only 25% of women population with AMH data available at baseline were selected to re-measure AMH at follow-up. However, the bias introduced by the selection of the participants may not affect our results much as the participants were selected randomly and their baseline characteristics, except for age, were not significantly different from those not included (Additional file 1: Table S1), but the small number of women included in this study is a concern. In addition, measuring AMH only at two time points of baseline and follow-up made it impossible to examine the longitudinal trajectory of AMH. Estimating rate of AMH decline based on the two measures need to be hypothesize a fixed pattern for AMH reduction in women during the follow-up although within-person variations in reduction of AMH over time have been indicated [39]. Moreover, although AMH is the best endocrine indicator of follicular reserve [1], the normative values for its yearly decline have not yet been defined. Consequently, we decided to use the highest tertile of the yearly rate of AMH decline as a cut point to define the rapid decline group. Since this study was conducted among Iranian women, our findings cannot be generalized to women from other populations that may have different dietary habits. In addition, our participants were a cohort of healthy women with regular menstrual cycles and with previously normal fertility, therefore our results cannot be generalized to women with infertility or those with PCOS [40]. We used blood samples that had not been collected on any specific days of menstrual cycles; however serum AMH levels are considered to be independent of menstrual phase [41].

## Conclusion

Our data demonstrates that dairy foods, carbohydrate, fat, protein, and calcium from dairy sources, lactose and galactose were inversely associated with the rate of decline in AMH and risk of its rapid decline. Considering the importance of rate of decline in AMH for both the reproductive and non-reproductive health of women, more prospective studies are required to elucidate the nutritional determinants of ovarian reserve reduction.

## Supplementary information

**Additional file 1: Table S1.** Characteristics of study participants.

**Additional file 2: Table S2.** Food groupings of food items.

## Data Availability

The dataset analyzed during the current study are available from the corresponding author on upon request.

## Abbreviations

AMH: Anti-Mullerian Hormone
BMI: Body mass Index
EIA: Enzyme Immunoassay
FFQ: Food Frequency Questionnaire
IQR: Interquartile range
MUFA: Mono-unsaturated Fatty Acids
PCOS: Polycystic Ovary Syndrome
PUFA: Poly-unsaturated Fatty Acids
SD: Standard Deviation
SFA: Saturated Fatty Acids
TLGS: Tehran Lipid and Glucose Study
USDA: United States Department of Agriculture

## References

[1] Dillon KE, Gracia CR (2013). What is normal ovarian reserve?. Semin Reprod Med.

[2] Jamil Z, Fatima SS, Ahmed K, Malik R (2016). Anti-Mullerian hormone: above and beyond conventional ovarian reserve markers. Dis Markers.

[3] Ramezani Tehrani F, Mansournia MA, Solaymani-Dodaran M, Steyerberg E, Azizi F (2016). Flexible parametric survival models built on age-specific antimullerian hormone percentiles are better predictors of menopause. Menopause.

[4] Iwase A, Osuka S, Goto M, Murase T, Nakamura T, Takikawa S (2018). Clinical application of serum anti-Mullerian hormone as an ovarian reserve marker: a review of recent studies. J Obstet Gynaecol Res.

[5] Freeman EW, Sammel MD, Lin H, Boorman DW, Gracia CR (2012). Contribution of the rate of change of antimullerian hormone in estimating time to menopause for late reproductive-age women. Fertil Steril.

[6] Amanvermez R, Tosun M (2016). An update on ovarian aging and ovarian reserve tests. Int J Fertil Steril.

[7] Gohari MR, Ramezani Tehrani F, Chenouri S, Solaymani-Dodaran M, Azizi F (2016). Individualized predictions of time to menopause using multiple measurements of antimullerian hormone. Menopause.

[8] de Kat AC, Verschuren WM, Eijkemans MJ, Broekmans FJ, van der Schouw YT (2017). Anti-Mullerian hormone trajectories are associated with cardiovascular disease in women: results from the Doetinchem Cohort Study. Circulation.

[9] Ramezani Tehrani F, Montazeri SA, Khalili D, Cheraghi L, Broekmans FJ, Momenan AA (2016). Age-specific anti-Mullerian hormone and electrocardiographic silent coronary artery disease. Climacteric..

[10] Richardson MC, Guo M, Fauser BC, Macklon NS (2014). Environmental and developmental origins of ovarian reserve. Hum Reprod Update.

[11] Pelosi E, Simonsick E, Forabosco A, Garcia-Ortiz JE, Schlessinger D (2015). Dynamics of the ovarian reserve and impact of genetic and epidemiological factors on age of menopause. Biol Reprod.

[12] Shahrokhi SZ, Kazerouni F, Ghaffari F (2018). Anti-Mullerian hormone: genetic and environmental effects. Clin Chim Acta.

[13] Moslehi N, Mirmiran P, Tehrani FR, Azizi F (2017). Current evidence on associations of nutritional factors with ovarian reserve and timing of menopause: a systematic review. Adv Nutr.

[14] Anderson C, Mark Park YM, Stanczyk FZ, Sandler DP, Nichols HB (2018). Dietary factors and serum antimullerian hormone concentrations in late premenopausal women. Fertil Steril.

[15] Azizi F, Ghanbarian A, Momenan AA, Hadaegh F, Mirmiran P, Hedayati M (2009). Prevention of non-communicable disease in a population in nutrition transition: Tehran Lipid and Glucose Study phase II. Trials.

[16] Azizi F, Zadeh-Vakili A, Takyar M (2018). Review of rationale, design, and initial findings: Tehran Lipid and Glucose Study. Int J Endocrinol Metab.

[17] Tehrani FR, Solaymani-Dodaran M, Tohidi M, Gohari MR, Azizi F (2013). Modeling age at menopause using serum concentration of anti-mullerian hormone. J Clin Endocrinol Metab.

[18] Executive summary of the clinical guidelines on the identification, evaluation, and treatment of overweight and obesity in adults. Arch Intern Med. 1998;158:1855–67. 10.1001/archinte.158.17.1855 9759681

[19] Mirmiran P, Esfahani FH, Mehrabi Y, Hedayati M, Azizi F (2010). Reliability and relative validity of an FFQ for nutrients in the Tehran lipid and glucose study. Public Health Nutr.

[20] Esfahani FH, Asghari G, Mirmiran P, Azizi F (2010). Reproducibility and relative validity of food group intake in a food frequency questionnaire developed for the Tehran Lipid and Glucose Study. J Epidemiol.

[21] Azar M, Sarkisian E. Food composition table of Iran. National Nutrition and Food Research Institute of Shaheed Beheshti University. (in Farsi).

[22] Ars.usda.gov (homepage on internet). Washington DC: United States Department of Agriculture, Agriculture of Research Service. [Updated 2009 April 27]. Available from: http://www.nal.usda.gov/fnic/foodcomp/.

[23] Dolleman M, Verschuren WM, Eijkemans MJ, Dolle ME, Jansen EH, Broekmans FJ (2013). Reproductive and lifestyle determinants of anti-Mullerian hormone in a large population-based study. J Clin Endocrinol Metab.

[24] Jung S, Allen N, Arslan AA, Baglietto L, Brinton LA, Egleston BL (2017). Demographic, lifestyle, and other factors in relation to antimullerian hormone levels in mostly late premenopausal women. Fertil Steril.

[25] Moslehi N, Shab-Bidar S, Ramezani Tehrani F, Mirmiran P, Azizi F (2018). Is ovarian reserve associated with body mass index and obesity in reproductive aged women?. A Meta-Analysis Menopause.

[26] Sjaarda LA, Schisterman EF, Schliep KC, Plowden T, Zarek SM, Yeung E (2015). Dietary carbohydrate intake does not impact insulin resistance or androgens in healthy. Eumenorrheic Women J Clin Endocrinol Metab.

[27] Nagel G, Altenburg HP, Nieters A, Boffetta P, Linseisen J (2005). Reproductive and dietary determinants of the age at menopause in EPIC-Heidelberg. Maturitas.

[28] Carwile JL, Willett WC, Michels KB (2013). Consumption of low-fat dairy products may delay natural menopause. J Nutr.

[29] Kim K, Wactawski-Wende J, Michels KA, Plowden TC, Chaljub EN, Sjaarda LA (2017). Dairy food intake is associated with reproductive hormones and sporadic anovulation among healthy premenopausal women. J Nutr.

[30] Chavarro JE, Rich-Edwards JW, Rosner B, Willett WC (2007). A prospective study of dairy foods intake and anovulatory infertility. Hum Reprod.

[31] Swartz WJ, Mattison DR (1988). Galactose inhibition of ovulation in mice. Fertil Steril.

[32] Bandyopadhyay S, Chakrabarti J, Banerjee S, Pal AK, Goswami SK, Chakravarty BN (2003). Galactose toxicity in the rat as a model for premature ovarian failure: an experimental approach readdressed. Hum Reprod.

[33] Cramer DW, Harlow BL, Barbieri RL, Ng WG (1989). Galactose-1-phosphate uridyl transferase activity associated with age at menopause and reproductive history. Fertil Steril.

[34] Fridovich-Keil JL, Gubbels CS, Spencer JB, Sanders RD, Land JA, Rubio-Gozalbo E (2011). Ovarian function in girls and women with GALT-deficiency galactosemia. J Inherit Metab Dis.

[35] Wise LA, Wesselink AK, Mikkelsen EM, Cueto H, Hahn KA, Rothman KJ (2017). Dairy intake and fecundability in 2 preconception cohort studies. Am J Clin Nutr.

[36] Nagata C, Takatsuka N, Kawakami N, Shimizu H (2000). Association of diet with the onset of menopause in Japanese women. Am J Epidemiol.

[37] Pearce K, Tremellen K (2016). Influence of nutrition on the decline of ovarian reserve and subsequent onset of natural menopause. Hum Fertil (Camb).

[38] Dorjgochoo T, Kallianpur A, Gao YT, Cai H, Yang G, Li H (2008). Dietary and lifestyle predictors of age at natural menopause and reproductive span in the Shanghai Women’s Health Study. Menopause.

[39] de Kat AC, van der Schouw YT, Eijkemans MJ, Herber-Gast GC, Visser JA, Verschuren WM (2016). Back to the basics of ovarian aging: a population-based study on longitudinal anti-Mullerian hormone decline. BMC Med.

[40] Tehrani FR, Solaymani-Dodaran M, Hedayati M, Azizi F (2010). Is polycystic ovary syndrome an exception for reproductive aging?. Hum Reprod.

[41] Tsepelidis S, Devreker F, Demeestere I, Flahaut A, Gervy C, Englert Y (2007). Stable serum levels of anti-Mullerian hormone during the menstrual cycle: a prospective study in normo-ovulatory women. Hum Reprod.

