# Supplementary material for: Do dietary intakes influence the rate of decline in anti-Mullerian hormone among eumenorrheic women? A population-based prospective investigation
Source: Nutr J. 2019 Dec 2;18:83. doi: 10.1186/s12937-019-0508-5 (PMC6889581; doi:10.1186/s12937-019-0508-5)
Supplement: Supplementary file 1 — Additional file 1: Table S1. Characteristics of study participants. [file 12937_2019_508_MOESM1_ESM.docx]

*Supplementary table 1. Characteristics of study participants ^1^*

| **Variables** | **Included** | **Not-included** | **p-value** |
| --- | --- | --- | --- |
| Number | 227 | 788 | - |
| Baseline age (years) | 37.2±6.33 | 35.7±7.36 | 0.005 |
| Housewife (n(%)) | 183 (80.6) | 634(80.5) | 0.521 |
| Education >12 years (n(%)) | 132 (58.1)^2^ | 399(50.6)^3^ | 0.084 |
| Never smokers (n(%)) | 219 (96.5) | 758(96.2) | 0.390 |
| Baseline BMI (kg/m2) | 26.9±4.32 ^4^ | 27.1±4.64^5^ | 0.511 |
| Baseline BMI categories |  |  |  |
| ≤25 (n(%)) | 74 (32.6) | 272(34.9) |  |
| 25-29.9 (n(%)) | 104 (45.8) | 303(38.9) | 0.106 |
| ≥30 (n(%)) | 47 (20.7) | 204(26.2) |  |
| Age at menarche (years) | 13.5±1.37^6^ | 13.4±1.46^7^ | 0.634 |
| **Dietary intakes** |  |  |  |
| Energy (kcal/day) | 2274±688 | 2340±766^8^ | 0.425 |
| Carbohydrate (% of energy) | 58.1±5.94 | 57.9±5.90^8^ | 0.730 |
| Fat (% of energy) | 30.9±5.26 | 31.1±5.80^8^ | 0.425 |
| Protein (% of energy) | 13.5±2.16 | 14.7±2.65^8^ | 0.704 |

*^1^Data are presented as mean ± standard deviation (SD), median (quartile 1, quartile 4), and Number (%).*

*^2^Available for n=222.*

*^3^ Available for n=767.*

*^4^Available for n=225.*

*^5^ Available for n=779.*

*^6^Available for n=226.*

*^7^Available for n=702.*

*^8^Available for n=622.*
