# Supplementary material for: Do dietary intakes influence the rate of decline in anti-Mullerian hormone among eumenorrheic women? A population-based prospective investigation
Source: Nutr J. 2019 Dec 2;18:83. doi: 10.1186/s12937-019-0508-5 (PMC6889581; doi:10.1186/s12937-019-0508-5)
Supplement: Supplementary file 2 — Additional file 2: Table S2. Food groupings of food items. [file 12937_2019_508_MOESM2_ESM.docx]

**Supplementary Table.** Food groupings of food items

| **Food groups** | **Food items** |
| --- | --- |
| Grains | Dark bread (Sangak, Barbari, Taftoon), White bread (Lavash , baguette), toasted bread, , noodles, pasta, rice, cooked barely, bulgur |
| Legumes | All kind of legumes including beans, peas, lima beans, lentils, soy |
| Total meats | Beef, lamb, poultry, fish, organ meat |
| Beef-lamb | Beef, lamb |
| Poultry | Chicken |
| Fish | Different kinds of fresh fish |
| Tuna fish | Canned tuna fish |
| Organ meats | Organ meats of chicken, lamb and beef |
| Dairy products | Milk, yoghurt, cheese (both low and high fat),chocolate milk, doogh (yoghurt drink), cream, kashk, ice cream |
| Milk | Low fat and high fat milk, chocolate milk |
| Fermented dairy | Yoghurt, cheese, Dough, Kashk, ice cream |
| Vegetables | Cruciferous vegetables, tomatoes , carrots, cucumbers, eggplants, corn, garlic, turnips, squash, mushrooms, onions, pumpkin, celery, green peas, green beans, green paper, spinach, lettuce, mixed vegetables, shallot |
| Allium | Onions, garlic, shallot |
| Cruciferous | Cabbage, cauliflower, Brussels sprouts and broccoli |
| Potatoes | Boiled and fried potato |
| Green vegetables | Lettuce, Mixed green vegetables, cucumbers, spinach, Bell paper, green bean, celery, green summer squash |
| Yellow/orange vegetables | Pumpkin, carrot |
| Fruits | All fresh fruits, dried fruits |
| Fruit juices | Apple juice, orange juice, other fruit juices |
| Dried fruits | Figs, mulberries, prunes, apricots, peaches, raisins |
| Melons | Different kinds of melons |
| Stone fruits | Nectarines, apricots, peaches and plums |
| Citrus fruits | Oranges, grapefruits, mandarins and limes |
| Berries | Cherry, strawberries, kiwifruit, mulberry, grapes |
| Nuts | Peanuts, almonds, walnuts, pistachios, hazelnuts, roasted seeds |
| Olive -olive oil | Olive and olive oil |
| Oil-fats | Vegetable oils, Hydrogenated vegetable fat, animal fats |
| Butter | Butter, margarine, and mayonnaise |
| Fast foods | Pizza, processed meats, hamburger, French fries |
| Sweets and cakes | Sugar, cube sugar, chocolates, candies and nabat, traditional sweets(gaz, noghle, souhan, halva ), Biscuits, cakes, cookies |
| Tea and coffee | Tea, coffee |
| Salty snacks | Potato chips, puffs, crackers |
